# Public and Patient Involvement in Research in the Fields of Inflammatory or Autoimmune Ocular Diseases: Protocol for a Scoping Review

**DOI:** 10.2196/88991

**Published:** 2026-08-21

**Authors:** Gráinne Tynan, Deirdre Collins, Eileen Sheehy, Monika Lauder, Nikki Dunne, Emily Greenan, Killian Walsh, Andrea Doyle, Joan Ní Gabhann-Dromgoole

**Affiliations:** 1 Sjögren’s Ireland, Dublin, Leinster, Ireland; 2 School of Pharmacy and Biomolecular Sciences, RCSI, University of Medicine and Health Sciences, 123 St Stephen's Green, Dublin, Leinster, D2, Ireland, 353 014025216; 3 Royal Victoria Eye & Ear Hospital Ireland, Irish College of Opthalmologists, Dublin, Leinster, Ireland; 4 RCSI Library, RCSI, University of Medicine and Health Sciences, Dublin, Leinster, Ireland; 5 SIM Centre for Simulation Education and Research, RCSI, University of Medicine and Health Sciences, Dublin, Leinster, Ireland

**Keywords:** patient participation, patient and public involvement, autoimmune diseases, eye diseases, inflammation, scoping review, research design

## Abstract

**Background:**

Patient and public involvement (PPI) is increasingly recognized as an important component of inclusive and patient-centered health research. However, the extent and nature of PPI in autoimmune and inflammatory ocular disease research remain unclear and inconsistently reported.

**Objective:**

This scoping review aims to map how patients and the public have been involved in research relating to autoimmune and inflammatory ocular diseases across clinical, translational, and preclinical domains.

**Methods:**

This scoping review follows the methodological framework proposed by Arksey and O’Malley and refined by Levac, while incorporating elements of JBI guidance for scoping reviews. Reporting is informed by the PRISMA-ScR (Preferred Reporting Items for Systematic Reviews and Meta-Analyses extension for Scoping Reviews) checklist. The MEDLINE, Embase, and CINAHL databases were searched from inception to March 2025. Studies describing patient or public involvement in autoimmune or inflammatory ocular disease research were considered eligible for inclusion. Screening, data extraction, and thematic synthesis will be conducted using predefined eligibility criteria and a prepiloted charting form.

**Results:**

Database searches were completed in March 2025. Following piloting of eligibility criteria and screening procedures, title and abstract screening commenced in April 2025. Full-text screening, data charting, and synthesis were planned sequentially in accordance with the protocol described, with completion of the review anticipated in December 2026. Findings from the completed scoping review will be disseminated through peer-reviewed publication, conference presentations, and patient advocacy networks.

**Conclusions:**

This scoping review will provide the first comprehensive overview of how PPI has been implemented within autoimmune and inflammatory ocular disease research. Mapping current approaches and gaps may help support more meaningful integration of patient perspectives in future research and strengthen cocreation approaches within ophthalmology and autoimmune disease research.

## Introduction

Autoimmune and inflammatory ocular diseases encompass a heterogeneous group of conditions affecting both ocular and systemic health, including Sjögren disease, thyroid eye disease, uveitis, and chronic inflammatory ocular surface disorders [1-3]. These conditions are frequently associated with a substantial symptom burden, including ocular pain, fatigue, visual disturbances, and reduced quality of life [4,5]. Patients often experience delayed diagnosis, fragmented care pathways, and limited access to disease-specific information, particularly in conditions such as Sjögren disease where symptoms may precede diagnosis by several years [1,6-8].

Sjögren disease in particular is a chronic and frequently underdiagnosed autoimmune condition affecting approximately 0.1% to 0.6% of the population and disproportionately affecting women [5,9]. It is characterized by a broad range of systemic and ocular manifestations, including dry eye disease, joint pain, fatigue, glandular dysfunction, and neuropsychiatric symptoms, and it can substantially affect the quality of life [5,7,9,10]. As a condition with significant ocular involvement and well-documented patient burden, Sjögren disease provides an important example of the challenges experienced across autoimmune and inflammatory ocular diseases more broadly.

Despite increasing recognition of these impacts, research priorities have often focused predominantly on measurable clinical end points or molecular mechanisms, with comparatively less attention given to patient-reported experiences, functional outcomes, and treatment burden [11,12]. This disconnect contributes to growing international calls for more meaningful patient and public involvement (PPI) in health research. PPI is increasingly acknowledged not just as a mechanism for improving relevance and transparency but as a core element of ethical, inclusive research practice [13,14].

Global initiatives such as INVOLVE (United Kingdom), SPOR (Strategy for Patient-Oriented Research; Canada), and EUPATI (European Patients’ Academy on Therapeutic Innovation; European Union) have developed frameworks for embedding PPI throughout the research cycle. In Ireland, the PPI Ignite Network has expanded training, infrastructure, and guidance to foster collaborative partnerships between researchers and the public [15]. Evidence suggests that PPI contributes to more impactful research, greater participant trust, and improved uptake of findings [16-18]. Nevertheless, systematic reviews show that PPI is inconsistently reported and rarely critically evaluated, particularly in ophthalmology, immunology, and autoimmunity [19-21].

A scoping review methodology was selected because the literature relating to PPI in autoimmune and inflammatory ocular disease research is emerging, heterogeneous, and inconsistently reported across clinical, translational, and preclinical contexts. For the purposes of this review, clinical research refers to patient-facing clinical studies, translational research refers to research bridging laboratory and clinical application, and preclinical research refers to laboratory-based or mechanistic research conducted prior to clinical testing. Scoping reviews are particularly suited to mapping broad areas of evidence, clarifying key concepts, identifying gaps in the literature, and examining how research practices are implemented across different settings [22-24]. This approach also enables inclusion of diverse study designs and methods, which is important when examining the varying ways in which PPI may be implemented and reported across biomedical and health research.

This scoping review was co-designed with patient advocates with lived experience of Sjögren disease to support alignment with patient priorities and real-world concerns. Patient partners contributed to the refinement of the research question, eligibility criteria, and overall methodological approach through an ongoing collaborative partnership. The review aims to identify and map how PPI has been implemented across clinical, translational, and preclinical research relating to autoimmune and inflammatory ocular diseases. Findings from this review may help inform future research practices and support more meaningful integration of patient perspectives within ophthalmology and autoimmune disease research.

## Methods

### Registration and Protocol Availability

The protocol for this scoping review is registered with the OSF Registries [25]. The protocol is reported in line with the PRISMA-ScR (Preferred Reporting Items for Systematic Reviews and Meta-Analyses extension for Scoping Reviews) reporting checklist (adapted for scoping review protocol; Checklist 1) [26].

### Review Team

The review team comprises pharmacy and biomedical researchers, simulation educators, and patient advocates with lived experience of Sjögren disease. This interdisciplinary team ensures methodological rigor, experiential relevance, and real-world applicability throughout the review.

### Study Design

This scoping review followed the methodological framework originally proposed by Arksey and O’Malley [22] and further refined by Levac et al [23]. Elements of JBI guidance for scoping reviews were also incorporated to strengthen reporting transparency and methodological rigor. The review is reported in accordance with the PRISMA-ScR checklist (Checklist 1) [26,27]. The review question guiding this scoping review is “How has patient and public involvement been implemented in clinical, translational, and preclinical research relating to autoimmune and inflammatory ocular diseases?”

### PPI Statement

This protocol was codeveloped through an ongoing researcher-patient partnership established in May 2021, using a cocreation through consultation approach [28]. Patient advocates contributed to defining the research question, refining eligibility criteria, and shaping the methodological approach. Previous collaborative activities included co-design of educational resources for health care professionals and the public, development of a dedicated Sjögren information webpage, and codevelopment of Ireland’s first PPI survey for people living with Sjögren disease to identify research and care priorities. These activities formed part of an ongoing researcher-patient partnership and cocreation program previously described by Doyle et al [7,28]. This foundation of sustained co-design ensures that the scoping review reflects real-world concerns, lived experience, and patient-defined priorities from its inception. Patient partners will also codevelop dissemination materials, including lay summaries and visual outputs for patient advocacy communities.

### Eligibility Criteria

For the purposes of this review, PPI is defined as the active involvement of patients, carers, advocacy groups, or members of the public in the design, conduct, analysis, or dissemination of research, rather than participation solely as research participants. Eligible forms of involvement will include consultation exercises, priority-setting activities, co-design approaches, advisory roles, collaborative partnerships, and coproduction activities. Studies describing participants solely as research participants, without evidence of involvement in shaping the research process will be excluded.

Peer-reviewed studies describing or evaluating PPI within clinical, translational, or preclinical research relating to autoimmune or inflammatory ocular diseases will be eligible for inclusion. Studies will be required to report explicit patient or public involvement in at least one stage of the research process, including research priority setting, study design, conduct, analysis, or dissemination. Gray literature, conference abstracts, editorials, and studies without clearly defined PPI components will be excluded.

### Information Sources and Search Strategy

A systematic search of the MEDLINE (via Ovid), Embase (via Ovid), and CINAHL databases was conducted to identify studies describing PPI in autoimmune and inflammatory ocular disease research. These databases were selected in consultation with an information specialist to ensure broad coverage of biomedical, clinical, and nursing literature relevant to the review question.

The search strategy combined controlled vocabulary terms (eg, MeSH and Emtree headings) and free-text keywords relating to PPI, autoimmune or inflammatory disease, and ocular or eye conditions. Search concepts were combined using Boolean operators (AND and OR), with search syntax adapted for each database. Truncation, phrase searching, and database-specific subject headings were used where appropriate to optimize retrieval.

Searches were conducted from database inception to March 2025. Searches were limited to English-language studies involving human participants. Reference lists of eligible studies will also be screened to identify additional relevant publications.

Gray literature was excluded because the review aimed to focus on peer-reviewed studies providing sufficiently detailed descriptions of PPI approaches and research processes. Full search strategies for all databases are provided in Multimedia Appendix 1.

Reporting of the search strategy was informed by relevant elements of the PRISMA-S (Preferred Reporting Items for Systematic Reviews and Meta-Analyses literature search extension) guidance for literature searches.

### Selection Process

Search results will be imported into Covidence for deduplication and screening. Prior to formal screening, the eligibility criteria and data charting form will be piloted on a purposive sample of records to ensure consistency in interpretation and application. Title and abstract screening will be conducted by one reviewer in keeping with the exploratory nature of scoping review methodology. Full-text screening will be undertaken independently by 2 reviewers, with disagreements resolved through discussion and consensus.

### Data Extraction and Charting

The data charting form was developed iteratively by the review team and refined following pilot extraction of a sample of studies. This iterative approach was consistent with the exploratory objectives of scoping review methodology.

Extracted information will include the author and year of publication, country and setting, disease focus, type of research, study design, participants involved in PPI activities, stage and type of involvement, reported PPI activities and approaches, and reported impacts, challenges, enabling factors, and reflections relating to PPI.

For the purposes of this review, stages of involvement will include activities relating to research priority setting, study design, conduct, analysis, interpretation, and dissemination.

Levels of involvement will be categorized using established PPI reporting and involvement frameworks, including INVOLVE and GRIPP2 (Guidance for Reporting Involvement of Patients and the Public) guidance [29], and classified as consultation, collaboration, or coproduction where sufficient detail is provided by study authors.

Data extraction will be conducted by 1 reviewer using the prepiloted charting form. Extracted data and categorization decisions will be reviewed collaboratively within the research team to support consistency and clarity. The final data charting form is provided in Multimedia Appendix 2.

### Data Synthesis and Thematic Synthesis

Extracted data will be synthesized using narrative and thematic synthesis approaches. Study characteristics and PPI activities will be summarized descriptively in tables. Narrative synthesis will be used to compare how PPI has been implemented across different autoimmune and inflammatory ocular disease contexts and across clinical, translational, and preclinical research domains.

Thematic synthesis will be conducted using a deductive-inductive approach. Initial coding will be informed by recognized PPI reporting frameworks, including GRIPP2 short form categories relating to aims of involvement, methods, stages of involvement, reported outcomes, and reflections on PPI processes. Additional inductive coding will be undertaken to identify emerging themes relating to challenges, enabling factors, impacts, and approaches to involvement across studies.

Themes will be iteratively reviewed and refined through discussion within the research team to support consistency of interpretation. Comparisons will be made across studies to explore similarities and differences in how PPI is implemented and reported across research domains and disease areas.

Consistent with scoping review methodology, the synthesis will aim to map and characterize patterns of PPI rather than formally evaluate intervention effectiveness. Formal critical appraisal will not be undertaken, consistent with the exploratory objectives of scoping review methodology.

### Ethical Considerations

As this scoping review involved analysis of published literature and did not involve collection of primary research data, formal research ethics committee approval was not required in accordance with institutional guidance for literature-based research. PPI contributors participated as research partners in the development of the protocol rather than as research participants. Contributors were involved in refining the research question, eligibility criteria, and dissemination planning through collaborative discussions and review meetings. No personal or sensitive data were collected from contributors for research purposes. Patient partners were supported in accordance with Royal College of Surgeons in Ireland (RCSI) PPI guidance, and reimbursement for involvement activities was provided through the RCSI PPI Ignite seed funding scheme.

## Results

Database searches were completed on March 3, 2025, and title and abstract screening commenced in April 2025 following piloting of the eligibility criteria and screening procedures. As of August 2026, full-text screening was underway. Data charting and synthesis will follow, with completion of the scoping review anticipated by December 2026. Findings will be disseminated through a peer-reviewed publication, conference presentations, and patient advocacy networks.

## Discussion

Autoimmune and inflammatory ocular diseases are associated with substantial symptom burden, diagnostic complexity, and significant impacts on quality of life. In conditions such as Sjögren disease, patients frequently report challenges relating to delayed diagnosis, fragmented care pathways, and limited access to disease-specific information [7,8,10]. These experiences highlight the importance of ensuring that research priorities align with the needs and concerns of those living with these conditions.

PPI provides an opportunity to strengthen the relevance, accessibility, and impact of health research. Systematic reviews demonstrate that PPI can enhance research transparency, prioritize real-world concerns, and improve uptake of findings [19-21]. However, within autoimmune and inflammatory ocular disease research, PPI remains inconsistently reported and is often poorly described, particularly within translational and preclinical research contexts [21]. This scoping review will provide a comprehensive overview of how patients have contributed to shaping research questions, methodologies, analysis, and dissemination in these fields.

This scoping review will provide a comprehensive overview of how PPI has been implemented and reported across clinical, translational, and preclinical research relating to autoimmune and inflammatory ocular diseases. Our approach builds on the exploratory scoping review framework proposed by Arksey and O’Malley [22] and refined by Levac et al [23], while incorporating JBI guidance and PRISMA-ScR reporting standards to support transparency and reproducibility [26,27]. The review also draws on a sustained patient-researcher partnership, with patient advocates contributing to the development of the research question, eligibility criteria, and methodological approach [22,23,28,30].

By mapping existing approaches to PPI across the research spectrum, this review may help inform future research practices, funding applications, and reporting standards. Findings may also be relevant to adjacent fields, including rheumatology, immunology, and neurology, where similar challenges relating to patient-centered research and stakeholder engagement exist. More broadly, this protocol demonstrates how sustained cocreation can contribute to the development of research that is both methodologically rigorous and responsive to patient priorities. Potential limitations of this review should also be acknowledged. Restricting inclusion to peer-reviewed literature may exclude relevant PPI activities reported in gray literature, organizational reports, or advocacy-led initiatives. Although the search strategy was developed iteratively to capture variation in PPI terminology, inconsistencies in reporting and terminology may still affect study identification. In addition, as this review aims to map and characterize existing approaches rather than formally evaluate effectiveness, conclusions regarding the impact of specific PPI strategies will be limited. These limitations are inherent to the exploratory objectives of scoping review methodology and will be considered when interpreting and reporting findings.

Dissemination of findings will include peer-reviewed publication, conference presentations, patient advocacy events, and codeveloped lay summaries shared through patient networks and social media platforms. Dissemination plans were developed collaboratively with patient partners to support accessibility and relevance to the wider autoimmune ocular disease community.

## Supplementary material

10.2196/88991 Multimedia Appendix 1 Search strategies for all databases.

10.2196/88991 Multimedia Appendix 2 Data charting form.

10.2196/88991 Checklist 1 PRISMA-ScR reporting checklist (adapted for scoping review protocol).

## Abbreviations

EUPATI: European Patients’ Academy on Therapeutic Innovation
GRIPP2: Guidance for Reporting Involvement of Patients and the Public
PPI: patient and public involvement
PRISMA-S: Preferred Reporting Items for Systematic Reviews and Meta-Analyses literature search extension
PRISMA-ScR: Preferred Reporting Items for Systematic Reviews and Meta-Analyses extension for Scoping Reviews
RCSI: Royal College of Surgeons in Ireland
SPOR: Strategy for Patient-Oriented Research
