# Supplementary material for: Public and Patient Involvement in Research in the Fields of Inflammatory or Autoimmune Ocular Diseases: Protocol for a Scoping Review
Source: JMIR Res Protoc. 2026 Aug 21;15:e88991. doi: 10.2196/88991 (PMC13496391; doi:10.2196/88991)
Supplement: Supplementary file 1 [file resprot-v15-e88991-s001.docx]

**Full Database Search Strategies**

**Pubmed**

| **Item** | **Details** |
| --- | --- |
| Date searched | 3 March 2025 |
| Date coverage | Database inception to 3 March 2025 |
| Limits applied | English language; human studies |

**Table S1. Free-text keyword search strategy for MEDLINE (via PubMed)**

| **Step** | **Search Query** | **Results** |
| --- | --- | --- |
| 1 | “community-based participatory research” OR “patient participation” OR “patient public involvement” OR “patient engagement” OR “patient partner*” OR “PPI” | 82,240 |
| 2 | “disease*” OR “condition*” OR “autoimmunity” OR “autoimmune disease*” OR “inflammation” OR “systemic inflammation” OR “systemic disease*” | 11,291,094 |
| 3 | #1 AND #2 | 28,047 |
| 4 | “eye” OR “ocular” | 65,124 |
| 5 | #3 AND #4 | 18 |

**Table S2. Controlled vocabulary and subject heading search strategy for MEDLINE (via PubMed)**

| **Step** | **Search Query** | **Results** |
| --- | --- | --- |
| 1 | "Community-Based Participatory Research"[Mesh] OR "Patient Participation"[Mesh] OR "patient public involvement" OR "patient engagement" OR "patient partner*" OR "PPI" | 89,677 |
| 2 | "Disease"[Mesh] OR "Autoimmunity"[Mesh] OR "Autoimmune Diseases"[Mesh] OR "Inflammation"[Mesh] OR disease* OR condition* OR autoimmunity OR autoimmune disease* OR inflammation OR inflammatory OR systemic inflammation OR systemic disease* | 11,666,356 |
| 3 | #1 AND #2 | 33,320 |
| 4 | "Eye"[Mesh] OR "Eye Diseases"[Mesh] OR eye OR eyes OR ocular | 80,534 |
| 5 | #3 AND #4 | 24 |

**Embase**

| **Item** | **Details** |
| --- | --- |
| Date searched | 3 March 2025 |
| Date coverage | Database inception to 3 March 2025 |
| Limits applied | English language; human studies |

### **Table S3. Free-text keyword search strategy for Embase**

| **Step** | **Search Query** | **Results** |
| --- | --- | --- |
| 1 | 'community-based participatory research' OR 'patient participation' OR 'patient public involvement' OR 'patient engagement' OR 'patient partner*' OR 'ppi' | 107,658 |
| 2 | 'disease*' OR 'condition*' OR 'autoimmunity' OR 'autoimmune disease*' OR 'inflammation' OR 'systemic inflammation' OR 'systemic disease*' | 16,511,064 |
| 3 | #1 AND #2 | 51,664 |
| 4 | 'eye' OR 'ocular' | 928,584 |
| 5 | #3 AND #4 | 720 |

### **Table S4. Controlled vocabulary and subject heading search strategy for Embase**

| **Step** | **Search Query** | **Results** |
| --- | --- | --- |
| 1 | 'community-based participatory research' OR 'patient participation' OR 'patient public involvement' OR 'patient engagement' OR 'patient partner*' OR 'ppi' OR 'participatory research'/exp OR 'patient participation'/exp OR 'contact examination'/exp | 123,363 |
| 2 | disease* OR 'condition*' OR 'autoimmunity' OR 'autoimmune disease*' OR 'inflammation' OR 'systemic inflammation' OR 'systemic disease*' OR 'diseases'/exp OR 'autoimmunity'/exp OR 'autoimmune disease'/exp OR 'inflammation'/exp | 32,235,889 |
| 3 | #1 AND #2 | 98,391 |
| 4 | 'eye' OR 'ocular' OR 'eyes' OR 'eye'/exp | 1,207,149 |
| 5 | #3 AND #4 | 1,433 |
| 6 | Remove MEDLINE duplicates | 544 |

## CINAHL

| **Item** | **Details** |
| --- | --- |
| Date searched | 3 March 2025 |
| Date coverage | Database inception to 3 March 2025 |
| Limits applied | English language; human studies |

### **Table S5. Free-text keyword search strategy for CINAHL**

| **Step** | **Search Query** | **Results** |
| --- | --- | --- |
| S1 | "community-based participatory research" OR "patient participation" OR "patient public involvement" OR "patient engagement" OR "patient partner*" OR "PPI" | 14,023 |
| S2 | "disease*" OR "condition*" OR "autoimmunity" OR "autoimmune disease*" OR "inflammation" OR "systemic inflammation" OR "systemic disease*" | 1,826,996 |
| S3 | S1 AND S2 | 4,230 |
| S4 | "Eye" OR "Ocular" | 79,484 |
| S5 | S3 AND S4 | 19 |

### **Table S6. Controlled vocabulary and subject heading search strategy for CINAHL**

| **Step** | **Search Query** | **Results** |
| --- | --- | --- |
| S1 | "community-based participatory research" OR "patient participation" OR "patient public involvement" OR "patient engagement" OR "patient partner*" OR "PPI" OR (MH "Patient Participation+") OR (MH "Contact Tracing") | 36,996 |
| S2 | "disease*" OR "condition*" OR "autoimmunity" OR "autoimmune disease*" OR "inflammation" OR "systemic inflammation" OR "systemic disease*" OR (MH "Disease+") OR (MH "Autoimmune Diseases+") OR (MH "Inflammation+") | 1,984,003 |
| S3 | S1 AND S2 | 10,268 |
| S4 | "Eye" OR "Eyes" OR "Ocular" OR (MH "Eye+") | 102,372 |
| S5 | S3 AND S4 | 47 |

**Note**: The search strategies were developed iteratively in consultation with an information specialist and adapted appropriately for each database platform using controlled vocabulary and free-text search terms. Searches were intentionally broad to maximise sensitivity for identifying studies describing patient and public involvement in autoimmune and inflammatory ocular disease research.
