# Supplementary material for: Public and Patient Involvement in Research in the Fields of Inflammatory or Autoimmune Ocular Diseases: Protocol for a Scoping Review
Source: JMIR Res Protoc. 2026 Aug 21;15:e88991. doi: 10.2196/88991 (PMC13496391; doi:10.2196/88991)
Supplement: Supplementary file 2 [file resprot-v15-e88991-s002.docx]

**Data Extraction and Charting Form**

The following charting form was developed iteratively in accordance with Joanna Briggs Institute guidance for scoping reviews and was piloted and refined by the review team prior to full data extraction.

| Author | Year | Country | Disease Focus | Type of Research (Clinical/Translational/Preclinical) | Participants Involved in PPI | Stage of PPI Involvement | Type of PPI Involvement | Outcomes and Reflections | Notes |
| --- | --- | --- | --- | --- | --- | --- | --- | --- | --- |
|  |  |  |  |  |  |  |  |  |  |
|  |  |  |  |  |  |  |  |  |  |
|  |  |  |  |  |  |  |  |  |  |
|  |  |  |  |  |  |  |  |  |  |
