# Supplementary material for: Public and Patient Involvement in Research in the Fields of Inflammatory or Autoimmune Ocular Diseases: Protocol for a Scoping Review
Source: JMIR Res Protoc. 2026 Aug 21;15:e88991. doi: 10.2196/88991 (PMC13496391; doi:10.2196/88991)
Supplement: Supplementary file 3 [file resprot-v15-e88991-s003.docx]

### **PRISMA-ScR Reporting Checklist (Adapted for Scoping Review Protocol)**

| **Section** | **Item** | **Description for Protocol** |
| --- | --- | --- |
| Title | 1 | Identifies the manuscript as a scoping review protocol: “Public and patient involvement in research in the fields of inflammatory or autoimmune ocular diseases: A scoping review protocol”. |
| Abstract | 2 | Structured summary includes Background, Objective, Methods, Results and Conclusions. |
| Introduction | 3 | Rationale provided regarding the need for examining patient and public involvement in autoimmune and inflammatory ocular disease research. |
| Introduction | 4 | Objectives stated: to identify and map how patient and public involvement has been implemented across clinical, translational and preclinical research contexts. |
| Methods | 5 | [The protocol for this scoping review was developed a priori and registered with the Open Science Framework (OSF) (https://osf.io/q2k4h/).](https://osf.io/q2k4h/) |
| Methods | 6 | Eligibility criteria provided, including inclusion and exclusion criteria relating to patient and public involvement activities and autoimmune or inflammatory ocular disease research. |
| Methods | 7 | Information sources described, including MEDLINE, Embase and CINAHL. |
| Methods | 8 | Search strategy described, including use of controlled vocabulary and free-text terms. Full database search strategies are provided in Supplementary Material 1. |
| Methods | 9 | Selection process described, including piloting procedures, single-reviewer title and abstract screening, dual-reviewer full-text review and use of Covidence software. |
| Methods | 10 | Data charting process described, including development and iterative refinement of the extraction form. |
| Methods | 11 | Data items described, including disease focus, research type, participants involved in PPI, stage and type of involvement, and reported outcomes and reflections. |
| Methods | 12 | Formal critical appraisal was not undertaken, consistent with the exploratory objectives of scoping review methodology. |
| Methods | 13 | Narrative and thematic synthesis approaches described, including deductive-inductive thematic analysis informed by GRIPP2 guidance. |
| Results | 14 | Study progress and screening status reported, including numbers of records screened and included. |
| Discussion | 15 | Discussion includes anticipated contribution of the review, methodological considerations and implications for future patient and public involvement research practices. |
| Funding | 16 | Funding sources and support statements included. |
